# Autoimmune hypothyroidism GWAS reveals independent autoimmune and thyroid-specific contributions and an inverse relation with cancer risk

**DOI:** 10.21203/rs.3.rs-4626646/v1

**Published:** 2024-07-08

**Authors:** Mary Reeve, Masahiro Kanai, Daniel Graham, Juha Karjalainen, Shuang Luo, Nikita Kolosov, Cameron Adams, Jarmo Ritari, Konrad Karczewski, Tuomo Kiiskinen, Zachary Fuller, Juha Mehtonen, Mitja Kurki, Zia Khan, Jukka Partanen, Mark McCarthy, Mykyta Artomov, Tiinamaija Tuomi, Matti Pirinen, Jukka Kero, Ramnik Xavier, Mark Daly, Samuli Ripatti, Finn Gen

**Affiliations:** Institute for Molecular Medicine Finland (FiMM); Broad Institute of MIT and Harvard; Broad Institute; Institute for Molecular Medicine Finland (FIMM), University of Helsinki; Institute for Molecular Medicine Finland (FIMM); Northwest Cancer Research Hospital; Genentech; Veripalvelu; Broad Institute; Institute for Molecular Medicine Finland; Broad Institute; University of Helsinki; Broad Institute of MIT and Harvard; Genentech; Finnish Red Cross Blood Service; Genentech; Nationwide Children’s Hospital; Helsinki University Hospital; University of Helsinki; Research Centre for Integrative Physiology and Pharmacology,Institute of Biomedicine, University of Turku, Turku; Broad Institute of MIT and Harvard; Broad Institute; Institute for Molecular Medicine Finland, FIMM, HiLIFE, University of Helsinki, Helsinki; FinnGen

## Abstract

The high prevalence of autoimmune hypothyroidism (AIHT) - more than 5% in human populations - provides a unique opportunity to unlock the most complete picture to date of genetic loci that underlie systemic and organ-specific autoimmunity. Using a meta-analysis of 81,718 AIHT cases in FinnGen and the UK Biobank, we dissect associations along axes of thyroid dysfunction and autoimmunity. This largest-to-date scan of hypothyroidism identifies 418 independent associations (p < 5×10^− 8^), more than half of which have not previously been documented in thyroid disease. In 48 of these, a protein-coding variant is the lead SNP or is highly correlated (r^2^ > 0.95) with the lead SNP at the locus, including low-frequency coding variants at *LAG3, ZAP70, TG, TNFSF11, IRF3, S1PR4, HABP2, ZNF429* as well as established variants at *ADCY7, IFIH1* and *TYK2*. The variants at *LAG3* (P67T), *ZAP70* (T155M), and *TG* (Q655X) are highly enriched in Finland and functional experiments in T-cells demonstrate that the *ZAP70*:T155M allele reduces T-cell activation. By employing a large-scale scan of non-thyroid autoimmunity and a published meta-analysis of TSH levels, we use a Bayesian classifier to dissect the associated loci into distinct groupings and from this estimate, a significant proportion are involved in systemic (i.e., general to multiple autoimmune conditions) autoimmunity (34%) and another subset in thyroid-specific dysfunction (17%). By comparing these association results further to other common disease endpoints, we identify a noteworthy overlap with skin cancer, with 10% of AIHT loci showing a consistent but opposite pattern of association where alleles that increase the risk of hypothyroidism have protective effects for skin cancer. The association results, including genes encoding checkpoint inhibitors and other genes affecting protein levels of PD1, bolster the causal role of natural variation in autoimmunity influencing cancer outcomes.

## Introduction

Hypothyroidism is estimated to affect at least 5% of individuals, though the high rate of undiagnosed cases may underestimate this number by a factor of two (Chiovato, Magri, and Carlé 2019), (Virta and Eskelinen 2011). In areas of the world with iodine sufficiency, the most common cause of hypothyroidism is Hashimoto’s disease, an autoimmune attack on the thyroid leading to reduced thyroid hormone production. As such, it constitutes the most common autoimmune disease (Conrad et al. 2023), though at the same time is considered vastly underdiagnosed considering the non-specific and gradual onset of the many clinical symptoms it causes. Early detection is key since treatment and supplementation with levothyroxine (synthetic thyroid hormone T4) can largely alleviate symptoms and prevent longer-term complications.

Given the well-established broad sharing of genetic risk factors across autoimmune diseases, a genetic study of hypothyroidism at scale would be expected to provide significant insights into autoimmunity, as well as, specific insights into thyroid disease that might aid in early detection and effective treatment before significant thyroid damage has taken place.

Population-wide biobank resources enable unique, large-scale integration of clinical information across medical domains. Here we take advantage of FinnGen, a project initiated in 2017, to integrate genome information with life medical history in 10% of the Finnish population to detect all treated cases of hypothyroidism (removing any major non-Hashimoto’s causes of hypothyroidism) and perform a GWAS of AIHT. Repeating the same definitions in the widely used UK Biobank resource, we perform and describe here a genome-wide analysis of more than 80,000 AIHT cases, more than twice the sample size of prior studies and yielding 418 hits compared to a maximum of 153 in recent studies (Saevarsdottir et al. 2020), (Kichaev et al. 2019).

## Results

### Phenotypic Definitions from Registry Data

To pursue a GWAS of AIHT first required phenotypic definitions to capture a large but specific set of autoimmune hypothyroidism. Unlike most autoimmune diseases, which require advanced care in specialty clinics or hospitals where diagnoses are well-recorded, many cases of AIHT, if diagnosed, will be detected in primary care and recognized in health registry data through continuous use of levothyroxine. To use such data, however, required close attention to the removal of individuals who were hypothyroid as a consequence of thyroid ablation due to Graves disease, thyroid cancer, thyroidectomy, and congenital thyroid disease (Methods). Removal of nearly 10,000 such individuals from the broadest definition resulted in 54,752 cases of AIHT in FinnGen R12, with a tally of 231 genome-wide significant hits. Confirming our hypothesis that such phenotypic restrictions would create a more homogeneous phenotype for genetics, the larger, less-specific GWAS of 64,082 individuals treated for hypothyroidism actually contained substantially fewer (204/231) genome-wide significant hits. Of note, we hypothesized that, while there is certainly a shared autoimmune component between Graves’ and Hashimoto’s, there might also be variants with opposite effects on thyroid function. Thus we intentionally focused the primary scan on AIHT and describe the alignment of the results with the analogous meta-analysis of Graves’ disease.

### Meta-analysis of autoimmune hypothyroidism

Having optimized the phenotypic definition in FinnGen, we then implemented a close phenotype analog in UK Biobank data (Methods), identified 26,966 cases, and ran a standard inverse variance weighted fixed-effects meta-analysis. Using a strict linkage disequilibrium (LD) based definition of independence (Methods), this meta-analysis (involving a total of 81,718 cases and 732,951 controls) produced a total of 417 independent genetic associations outside the MHC (Supplementary Table 1) and a series of highly significant associations within the MHC centered at previously reported common variants spanning the *DRB1-DQA1-DQB1* locus (Saevarsdottir et al. 2020). Even conservatively ascribing associations within 1 Mb to the same ‘locus’ indicates at least 280 distinct genomic regions associated with autoimmune hypothyroidism. As expected with the much greater sample size, this substantially adds to the previously documented association count of 153 (Kichaev et al. 2019)).

### Overview of the hits

In 48 of 417 associations, the lead variant is itself, or in very high LD (r^2^ > 0.95) with a protein-coding variant (Table 1a), and another 19 having a coding variant with 0.95 > r^2^ > 0.70 t. Among these are well-established common variants (e.g., *PTPN22, SH2B3, FUT2*), as well as lower frequency hypomorphic alleles (e.g., *TYK2, IFIH1*) known to be associated with many autoimmune diseases.16 of the associations are low-frequency variants that are highly enriched in Finns (from 4 to more than 100-fold) (Table 1b) − 12 of which are found only in the FinnGen GWAS because of their low frequency in UKBB. Notably, 6 of these 12 map onto coding variants noted above.

A total of 51 associations have a minor allele frequency of < 5% in Finland. Sixteen of these 51 (31.4%) are in the most likely ‘coding association’ (r^2^ > 0.95/Table 1) category, while only 32 of the 366 (8.7%) more common variants are - a significant (p < .0005) 3.5-fold excess which is the likely consequence of both selection (higher effect alleles being kept lower in frequency) and function (higher effect alleles detected in frequency-agnostic GWAS analysis are more often coding than lower effect ones).

The preponderance of associated coding variants at lower frequencies provides the most direct set of novel clues to disease biology. Noteworthy among these findings include a missense variant in *LAG3* (P67T), an inhibitory immune receptor for which inhibitors have been recently approved as immunotherapy in advanced melanoma (Tawbi et al. 2022; Long et al. 2023). No prior significant associations to coding variation in *LAG3* have been reported. The most significant of the novel Finnish associations, however, is a non-coding variant (chr16:27384341:C:CT) roughly 20 kb from the TSS of *IL21R*. Additionally, rare missense variants in both *IRF3* and *IRF4* confer protection from AIHT (with observed effect sizes greater than the protective hypomorphic missense variants at *TYK2* and *IFIH1*), and low-frequency risk variants of likely immune relevance are newly documented at *NFKBIZ* and *CD2*.

Two *PER3* variants in perfect linkage disequilibrium (P415A and H417R) were also among the implicated findings. While no prior immune-mediated disease links exist, this haplotype has been associated with morning chronotype in UKBB (Jones et al. 2019) and was earlier reported (Zhang et al. 2016) to segregate in a family with familial advanced sleep phase syndrome. In the latter work, this low-frequency haplotype was demonstrated to reduce PER3 protein levels and repressor activity.

### Hypomorphic mutation of *ZAP70* drives autoimmunity and immune deficiency

Another novel and Finnish-enriched association from this set is a missense variant in *ZAP70* (T155M), a tyrosine kinase essential for signal transduction downstream of the T-cell receptor (TCR) in response to antigen recognition. *ZAP70* is an established autosomal recessive cause of severe combined immunodeficiency (Arpaia et al. 1994; Elder et al. 1994; A. C. Chan et al. 1994) marked by an absence of CD8 + T-cells and CD4 + T-cells which do not respond to TCR-mediated activation. Given prior suggestion that *ZAP70* inhibition might be therapeutically efficacious in autoimmunity (Rao et al. 2021; Visperas et al. 2017), and the genetic parallel to *TYK2* where an allelic series involving distinct immunodeficiency and autoimmune protective alleles led to a recently approved therapeutic, we sought to further elucidate the function of this novel protective genetic association to autoimmune disease.

ZAP70 exists in an autoinhibited conformation at baseline, and upon productive TCR engagement, the two tandem SH2 domains of ZAP70 bind to phosphotyrosine motifs in CD3zeta associated with the TCR. This recruitment coincides with phosphorylation of ZAP70 in the interdomain B region and kinase domain by Lck, which elicits full activation of ZAP70, promoting phosphorylation of key substrates such as SLP76 and LAT that propagate the TCR signaling cascade (Ashouri et al. 2022).

**Figure 1.**
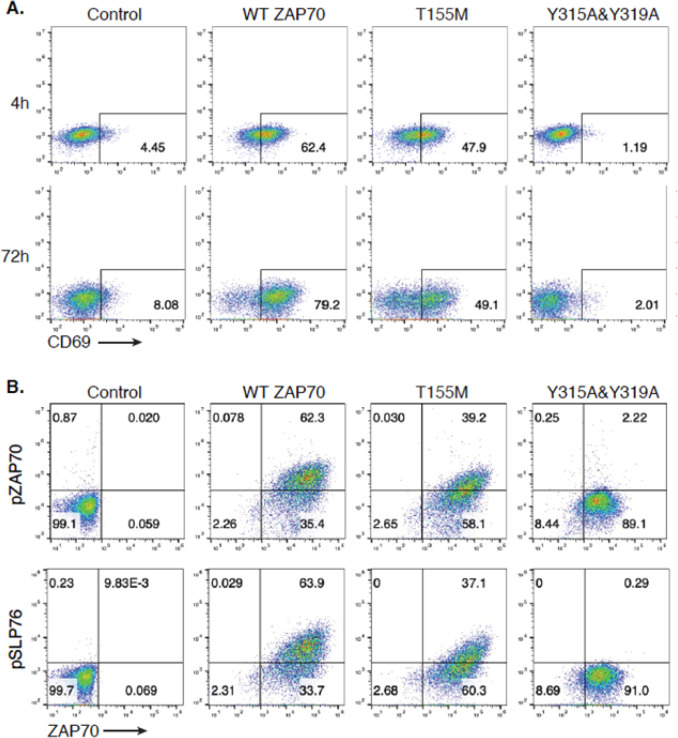
Impaired T cell activation in ZAP70 T155M variant T cell line in response to TCR stimulation. ZAP70-deficient Jurkat cells (P116 clone) were reconstituted with either ZAP70 or variants and stimulated with anti-CD3 and anti-CD28 (1ug/ml). Cells were then stained for detection of CD69 (**A**) or ZAP70, phospho-SLP76 (**B**) prior to FACS analysis. Control: ZAP70 deficient cells infected with control virus; WT ZAP70: ZAP70 deficient cells reconstituted with wide-type ZAP70; T155M: ZAP70 deficient cells expressing ZAP70 T155M; Y315A&Y319A: ZAP70 deficient cells expressing an inactive mutant ZAP70 Y315A&Y319A.

Several rare variants of *ZAP70* have been causally linked to Mendelian inheritance of a variety of immunopathologies that either ablate protein expression, impair kinase activity, or relieve autoinhibition (Sharifinejad et al. 2020). The clinical heterogeneity associated with these variants is complex, and the most common manifestations are combined immunodeficiencies associated with recurrent infections; although, a substantial subset of patients exhibit paradoxical autoimmunity and lymphoproliferative syndromes (Sharifinejad et al. 2020). We sought to determine how the T155M variant impacts ZAP70 function and whether it is associated with a gain of function through loss of autoinhibition or impaired function. Towards this end, we reconstituted ZAP70-deficient Jurkat T cells with variants of interest and monitored TCR signaling. WT ZAP70-reconstituted cells upregulated expression of the activation marker CD69 and induced phosphorylation of SLP76 and ZAP70 after TCR stimulation, whereas parental ZAP70-deficient cells did not (Fig. 1). Cells expressing a *ZAP70* double tyrosine mutant (Y315A&Y319A) within interdomain B, which is incapable of activation, showed a complete block of activation and SLP76 phosphorylation after TCR stimulation (Fig. 1). Cells reconstituted with *ZAP70* T155M exhibited a partial block in activation and phosphorylation of SLP76 (Fig. 1). Thus, the *ZAP70* T155M missense variant associated with autoimmunity impairs TCR signaling strength through an incomplete loss of function. Consistent with this observation, T155M also increases risk to immunodeficiencies in FinnGen (p < .0001).

### Intersection of hypothyroidism with checkpoint inhibition

PD-1 checkpoint inhibition to activate systemic immune responses has rapidly emerged as a critical tool in the cancer therapy arsenal, with considerable effort dedicated to understanding the underlying beneficial mechanisms, including enhancement of T-cell priming and activation and reinvigoration of exhausted intra-tumoral T-cells (Pauken and Wherry 2015; Oh et al. 2020). However, immune-related adverse events (irAEs), particularly new-onset autoimmune diseases such as hypothyroidism, type 1 diabetes, colitis, hepatitis, myocarditis, and vitiligo, remain an important clinical challenge in the use of these important drugs. Recent observations, (Freeman-Keller et al. 2016; Eggermont et al. 2020; Kotwal, Kottschade, and Ryder 2020; Khan et al. 2021), however, have demonstrated that individuals with irAEs may be receiving greater benefit from checkpoint inhibition, an observation confirmed in a recent meta-analysis (Hussaini et al. 2021). In one recent study, anti-PD-L1 atezolizumab-induced thyroid dysfunction was associated with longer survival across 7 trials in 6 cancer types. Furthermore, in one trial, patients with higher hypothyroid polygenic risk scores had both higher rates of atezolizumab-induced thyroid dysfunction and lower risk of death in triple-negative breast cancer (Khan et al. 2021).

The relationship between checkpoint inhibition and hypothyroidism in clinical practice, alongside observing individual associations to *CTLA4* and *LAG3*, encouraged us to look further at the role of genetic predisposition. Starting with the other two targets with approved drugs (PD-1 (encoded by *PDCD1*) and PD-L1 (encoded by *CD274*), we find the locus at *CD274* contains a nearby upstream genome-wide significant hit (rs911760) (along with a second independent hit at neighboring *PDCD1LG1*). Binding of CTLA-4 to CD80 and CD86 prevents continued T-cell activation, and among our strongest genome-wide significant associations, we observe variants in LD with the well-described *CT60* variant at *CTLA4* (rs3087243), which is correlated with increased CTLA-4 levels on CD4 + T-cells (Kasela et al. 2017) - consistent with tamping down immunity with consequent lowering of risk to AIHT (beta=−0.155, p = 2.7e-127). We further observe genome-wide significant associations at both *CD80* and *CD86*. Eleven variants spanning *CD80* (p = 4.3e-14) (and neighboring gene *TIMMDC1*) are associated with AIHT, and variants at the *ILDR1* and *CD86* locus are genome-wide significant (p = 4.4e-10). Collectively, these genetic findings suggest a strong relationship between the mechanism of checkpoint inhibition and reduced AIHT. The consistency of the genetic targets and allelic effects of these AIHT hits with the induced effects of checkpoint immunotherapy supports the idea that the irAEs commonly seen in checkpoint immunotherapy represent an on-target effect as suggested by trial studies (Khan et al. 2021).

Further to this intersection, we integrated recently published proteomics data (Sun et al. 2023), fine-mapped the GWAS hits of 1500 protein levels, and identified that 7 of our hypothyroid hits were significantly associated with PD-1 levels. In all seven cases, the allele that increased hypothyroid risk increased PD-1 levels with most in the subgroup that were associated with broad autoimmune disease risk and cancer protection described below (Supplementary Table 2) - consistent with excessive T-cell activation contributing to risk of autoimmunity and protection from cancer.

### Genetic dissection of hypothyroid risk

#### Autoimmunity component

As autoimmune hypothyroidism sits at the nexus between thyroid disease and autoimmunity and occurs at particularly high frequency, we hypothesized that insights into the underpinnings of both systemic autoimmunity and specific thyroid disease processes would be present. To explore this, we performed a similar meta-analysis of individuals with a nonthyroid-based autoimmune disease (Methods) (excluding anyone with any form of thyroid disease). This is hereafter referred to as ‘autoimmune nonthyroid (AInonT)’, and the joint FinnGen + UKBB meta-analysis had 70,570 cases compared to 741,401 controls. Unsurprisingly, there was considerable overlap between AInonT and AIHT scans, with 62 of the 417 index variants from AIHT showing a p < 1.2×10^− 4^ (.05/417), and 96 of 304 with p < .0024 (1/417) − 92 of 96 with the same direction of effect (Supp Table 3).

#### Thyroid-specific component

Distinct from autoimmunity, congenital hypothyroidism most often results from gene defects in thyroid development (agenesis or dysgenesis) or production of thyroid hormones (dyshormonogenesis), and have been explored in animal and cellular models (López-Márquez et al. 2021). Cross-referencing our GWAS with the Genomics England clinical panel for congenital hypothyroidism (https://panelapp.genomicsengland.co.uk/panels/31/download/34/) indicates independent common variation at six of these genes are associated to AIHT (Supplementary Table 4), providing pointers to the thyroid-specific effects in our scan.

Expanding to population-wide hormone production variability, thyroid-stimulating hormone (TSH) levels are broadly used in clinical settings to diagnose hypothyroidism. A recent publication (Zhou et al. 2020) provided a scan of population TSH levels in individuals with no known thyroid disease across multiple biobanks (not including FinnGen or UKBB) and uncovered 74 genome-wide significant associations to serum TSH levels. Summary statistics from this study, alongside our study, enable a larger-scale assessment of which of our associations arises from a direct impact on thyroid development or function. Our 417 AIHT index variants similarly show a highly significant excess of overlapping associations with 67 (at p < 1.2×10^− 4^) and 83 (at p < .0024) associated with TSH levels in the earlier study. Consistent with expectation, 80 of 83 overlaps show increasing AIHT risk corresponds to higher TSH levels.

**Figure 2:**
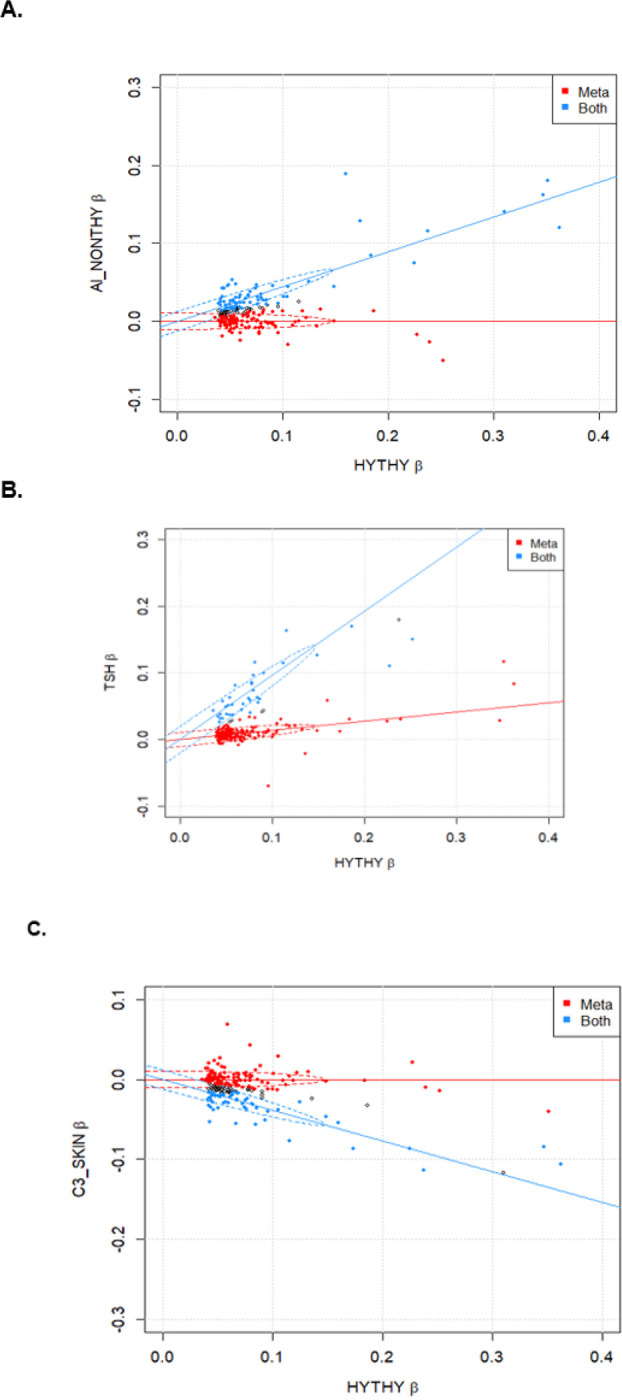
Scatter plots of effect sizes from AIHT-associated variant effect sizes compared with effect sizes from **A.** non-thyroid autoimmune diseases from FinnGen+UKBB, **B.** TSH levels from (Zhou et al.), and **C.** skin cancer from FinnGen+UKBB. Optimal two-group fit of Bayesian linemodels (Pirinen et al.) (run with parameters scale=0.6 and correlation=0.99) displayed with red indicating association to AIHT and blue indicating association to both AIHT and the query phenotype. Data points are colored if linemodels assignment with greater than 80% probability. Data points displayed (n=202) represent AIHT-independent index variants with p<1×10^−11^. (Full data and assignment probabilities listed in Supplementary Table 3)

To explore the shared effects between AIHT and AInonT and TSH in more detail, we applied linemodels, a Bayesian classification algorithm (Pirinen 2023), to compare the effect sizes of the 417 AIHT hits with those in the AInonT and TSH scans separately. We specifically ask whether a model in which there are two groups of variants (roughly ‘shared’ and ‘AIHT-specific’) fits the observed effect sizes better than a single relationship, and in the two-group case, assign group membership probabilities to each variant. Comparing AIHT to AInonT, a two-group solution (termed AIHT only and shared AI) was preferable to a one-group solution (p<10^−100^) with many variants assigned strongly to one or the other group (63 having > = 99% confidence of being shared, 68 having > = 99% confidence of being associated to AIHT only (Fig. 2, Supplementary Table 3). Running the same comparison between AIHT and TSH summary statistics (Zhou et al. 2020) produced an even more tail-heavy posterior assignment probability distribution among two groups (again preferred strongly over one group (p<10^−100^), with 37 having > 99% confidence in the joint AIHT-TSH group, while 285 with 99% confidence in the ‘AIHT-only’ group. Of note, in this latter comparison, only 396/417 AIHT associations were included since several rare and Finnish-enriched variants were not tested in the TSH study.

Notably, the shared with AInonT and shared with TSH subgroups were significantly non-overlapping. The variants in the 99% shared AIHT-AInonT were completely distinct from the 99% shared AIHT-TSH group, with a formal Spearman’s correlation across all AIHT loci between the posterior probability of being shared with AInonT and shared with TSH (Supplementary Table 3) producing a rho of −0.29, p = 4.6×10^− 9^)). As less significant associations are less able to be assigned confidently to shared or non-shared classes, we estimated sharing proportions from the top half of associations (202 AIHT index variants with p < 1×10^− 11^) and observe 34% of associations are shared with AInonT and 18% shared with TSH (Fig. 2).

Confirming the functional distinction between these sets, we utilized the above-mentioned UKBB proteomics data where we observe 27 of our index variants are significantly associated (p < 5×10^− 8^) in trans to TSHB (a component of the TSH heterodimer) levels - demonstrating consistent allelic effects where higher TSHB corresponds to AIHT risk. 26 of the 27 are among the 37 > 99% confident AIHT-TSH shared group, while none were in the AIHT-AInonT group. This collective set of findings demonstrates that the genetic architecture of autoimmune hypothyroidism can be broadly dissected into several distinct independent contributing components - one of which represents processes shared across autoimmune diseases and one of which represents thyroid-specific functional contributors.

As noted earlier, autoimmune thyroid disease generally refers to both Hashimoto’s (hypothyroidism) and Graves’ (hyperthyroidism). We utilized the FinnGen + UKBB meta-analysis of Graves’ disease (Methods) which had a total sample of 6550 cases and 823242 controls. Despite the much smaller sample, there was, as expected, a highly significant overlap with AIHT. Among the 417 AIHT index variants were 56 (at p < 1.2×10^− 4^) and 101 (at p < .0024) at the thresholds where .05 and 1 expected by chance. These associations were, however, not unidirectional, with 86 being shared and 15 in the opposite direction. This distinction, however, falls along and reinforces the same dimension described in the linemodels analysis. Specifically, 10 of the 15 loci where Hashimoto’s and Graves’ have opposite direction effects are in the 99% AIHT-TSH shared group, with 0 in the 99% shared AI (AIHT-AInonT) group, while among the 86 shared direction variants, 22 were in the 99% shared AI group with 0 in the AIHT-TSH shared group. Thus the two common forms of autoimmune thyroid disease appear to tightly share their autoimmune component, while their thyroid-specific component is also in common but acts in opposite directions of risk and protection in the two diseases.

### Inverse genetic risk shared with skin cancer

While the relationships to autoimmunity and thyroid function are not surprising, we sought to use the comprehensive phenotypic association in FinnGen to explore the potential overlap with other common diseases via both overall assessments of common disease incidence with AIHT genetic risk, genetic correlation as well as by examination of coincident genome-wide significant loci. AIHT PRS was positively correlated (at a conservative threshold of p < 1e-5) with hundreds of FinnGen endpoints - unsurprisingly led by nearly all autoimmune and thyroid disease phenotypes. However, a significant negative relationship was observed for seven FinnGen cancer endpoints (Supplementary Table 5), led by the incidence of basal cell carcinoma (r=−0.07, p = 1.7e-17) and all skin cancers (r = −0.06, p = 8.3e-13) with less significant negative overlaps also seen for prostate cancer and the “all cancer” phenotype.

To explore this further, we then performed the analogous FinnGen + UKBB meta-analysis of skin cancer (including melanoma and non-melanoma − 68822 cases and 657740 controls) and examined results at the 417 AIHT index variants (Supplementary Table 3). In this set, there were 13 loci exceeding genome-wide significance, 26 at a comparison-wide level of significance (p < 1.2×10^− 4^) and a total of 48 at a level expected once by chance in 417 loci (p < .0024). Almost as striking as the excess itself, 24 of the 26 (and 42 of the 48) were instances where the effects on risk in skin cancer and hypothyroidism were in opposite directions.

We again utilized linemodels and identified an AIHT-only and AIHT-skin cancer shared group, the latter grouping with a negative slope indicating variants at which hypothyroid risk alleles correspond to skin cancer protective alleles, and assigned posterior probabilities of assignment to each group for all 417 loci. We then performed Spearman correlation between the probability membership in the AIHT-skin cancer shared group with the previously defined probabilities of AIHT-AInonT and AIHT-TSH shared groupings. The AIHT-skin cancer membership was positively correlated with AIHT-AInonT (rho = 0.22, p = 6.4×10^− 6^) and negatively correlated with AIHT-TSH sharing (rho=−0.25, p = 6.2×10^− 7^), indicating that the shared component of the genetic architecture of skin cancer and AIHT represents an immune program rather than one specific to the thyroid. Among the 15 variants with > 99% posterior assignment to both AInonT and skin cancer shared groups are well known missense variants in *PTPN22, TYK2, IFIH1, FUT2* and *CCDC88B*, as well as the low-frequency *ZAP70* and *IRF3* variants noted above, an intronic *FLT3* variant that prematurely truncates *FLT3*, and several other established immune-mediated disease loci at *CTLA4, BACH2* and *IL2RA*.

To confirm these relationships in independent samples, we performed an association study of a hypothyroidism polygenic risk score (PGS) from UKBB across all phenotypes in FinnGen (Methods). As expected, the PGS was strongly positively associated with AIHT (OR = 1.71, p < 1×10^− 300^) and numerous other autoimmune diseases, including Type 1 Diabetes (T1D_WIDE; OR = 1.46, p < 1*10^− 300^), Seropositive rheumatoid arthritis (RHEUMA_SEROPOS_STRICT; OR = 1.38, p = 6.5*10^− 143^), Vitamin B12 deficiency anemia (D3_ANAEMIA_B12_DEF; OR = 1.40, p = 7.3*10^− 105^), Vitiligo (L12_VITILIGO; OR = 1.36, p = 1.63*10^− 10^) (Supplementary Table 6). By contrast, strong negative association was found between the UKBB PGS and multiple cancer endpoints, led by basal cell carcinoma (OR = 0.91, p = 3.0×10^− 39^), all skin cancer (OR = 0.92, p = 8.4×10^− 36^) and the umbrella ‘all cancer’ endpoint (OR = .96, p = 6.0×10^− 26^) as well as individually significant breast and prostate cancer endpoints. (Supplementary Table 7) Results were robust to two additional analyses, one of which removed the MHC from PGS calculation and evaluation, and a second which removed all FinnGen AIHT cases (to confirm independence from the diagnosed phenotype self-evidently related to the PGS). Naturally, this second analysis removes significant relationships to thyroid-related phenotypes but leaves the cancer and other autoimmune disease relationships intact (Supplementary Tables 8 and 9).

## Discussion

Using two large-scale biobanks with broad diagnostic and medication information, FinnGen and the UK Biobank, we present here the largest genome-wide association study to date in autoimmune hypothyroidism. Taking advantage of the extensive data available in these biobanks, including prescription medication use, provided a more complete ascertainment of cases, while the diagnostic coverage in these resources permitted the exclusion of other common thyroid conditions. The resultant analysis included a total of 81,718 cases and yielded a total of 417 independent genome-wide significant variants in addition to the MHC, roughly doubling the numbers found in the largest previous studies (Kichaev et al. 2019; Saevarsdottir et al. 2020).

Sixty-seven of these 417 associations (16%) contained coding variants that were, or were in high LD with, the lead variant. As coding variants were twice as often found in low-frequency association credible sets, these provided the most interpretable pointers to novel biological insights into AIHT. Among the coding variants likely driving associations were low-frequency coding variants in both *IRF3* and *IRF4*, which, similar to the hypomorphic low-frequency variants in *IFIH1* and *TYK2* also seen here, broaden the set of disease protective perturbations likely acting through interferon response. This connection is further supported by a common missense variant at *TLR3*, another interferon-inducing component of antiviral immunity, and by a common missense variant in the interferon-inducible *IFITM2*. Another notable association at *PER3* connects circadian regulation to AIHT via two tightly linked missense variants previously linked to morning chronotype and now demonstrated to be protective from AIHT.

We characterize the function of another of these coding variants where we find that *ZAP70*:T155M demonstrates a unique profile in that it is associated with a partial loss of function and autoimmunity, while more complete loss of function hypomorphic alleles homozygous produce severe combined immunodeficiencies (Sharifinejad et al. 2020). Additional complex *ZAP70* genotypes associated with autoimmunity have been discovered. For example, a compound heterozygous individual harboring both loss of function *ZAP70* R192W variant and gain of function R360P variant led to autoimmunity, but the combined effects of both alleles were required to precipitate disease (A. Y. Chan et al. 2016). Taken collectively, human genetics evidence suggests *ZAP70* function must be optimized within a narrow range such that partially impaired activity or enhanced activity elicits autoimmunity (Ashouri et al. 2022). Mouse models have been absolutely indispensable in conclusively demonstrating this concept and establishing the mechanism of action. Hypomorphic *ZAP70* alleles generated from a chemical mutagenesis screen in mice were shown to impair TCR signaling strength, resulting in altered thymocyte development and selection of an autoreactive TCR repertoire associated with the development of dsDNA antibodies and hyper-IgE syndrome (Siggs et al. 2007). Similarly, a spontaneously arising point mutation of *ZAP70* in SKG mice led to a partial loss of function and impaired negative selection of autoreactive T cells resulting in arthritis (Sakaguchi et al. 2003). Adoptive transfer of naïve SKG T cells into immunocompromised recipients was sufficient to induce arthritis, suggesting impaired central and peripheral tolerance (Ashouri et al. 2019). The *ZAP70* T155M variant associated with autoimmune thyroid disease appears to act through a similar mechanism of impaired TCR signaling resulting in loss of tolerance. This is likely to occur through combined effects of impaired negative selection of autoreactive T cells, lymphopenia-induced homeostatic expansion of pathogenic T cells, defective Treg development/function, and resistance to peripheral tolerance mechanisms such as Treg suppression or anergy induction (Liston, Enders, and Siggs 2008). These findings have important implications for the treatment of autoimmunity. Having demonstrated that the direction of effect for the *ZAP70* T155M risk variant is a partial loss of function, targeting *ZAP70* kinase activity with inhibitors to treat autoimmunity could come with unanticipated consequences. Complete inhibition of *ZAP70* may ameliorate T cell-driven autoimmunity at the expense of immunodeficiency, whereas partial inhibition of *ZAP70* may exacerbate dysregulation of self-tolerance.

The sizable number of GWAS hits in this scan enabled us to not only detect significant polygenic overlaps with other phenotypes such as non-thyroid autoimmune diseases and TSH levels (neither of which is individually surprising) but also enable the determination that these particular overlaps make up distinct, non-overlapping components of AIHT risk. Through the use of Bayesian linemodels, we estimate 34% of AIHT associations are shared with autoimmune diseases more broadly, and 18% are shared with variation that elevates TSH levels unrelated to immunity. The parallel analysis of Graves’ disease and AIHT indicates that while there is widespread same-direction sharing of the autoimmune components, the thyroid/TSH alleles act in opposite directions consistent with the hyper versus hypothyroid character of each disease.

PRS analysis demonstrated a correlation of AIHT PRS to lower risk of skin, as well as breast and prostate cancer. Considerable sharing between the AIHT and skin cancer GWAS indicated that the opposite effect alleles conferring risk to AIHT and protection from skin cancer were concentrated in the autoimmune component of AIHT rather than being thyroid function related. While skin cancer, and particularly basal cell carcinoma, showed uniquely strong opposite direction effects, the same highly significant observation in prostate and breast cancer suggests this is most likely a consequence of general immune surveillance and response to emergent solid tumors, which, while likely of differential relevance to different tumor types, is not specific to skin.

Further to this connection, the results also flag genetic variation associated with AIHT implicating the majority of genes involved in successful targets of checkpoint immunotherapy, including a novel rare coding variant in *LAG3*. In addition, seven AIHT risk alleles are significantly associated with PD-1 levels in recently published proteomic data from the UK Biobank. Moreover, the genetic intersection between AIHT risk and protection from skin cancer further sheds light on published observations that individuals with irAEs receive greater benefits from checkpoint immunotherapy. Demonstration that the same hypothyroid genetic risk that predisposes to thyroid irAEs and improved immunotherapy outcomes represents a general population-wide signature of cancer protection suggests naturally arising genetically mediated variation in immune surveillance or function, partially encoded in the same checkpoint pathway, is a significant contributor to inter-individual variation in cancer risk and potentially points to mechanisms that could be effective in prevention as well as treatment.

## Methods

### Study Cohort

The FinnGen study (https://www.finngen.fi/en) is a public-private partnership founded in 2017, including Finnish universities, biobanks, and hospital districts, as well as several pharmaceutical companies. The aim is to collect both National Health Records and genetic data from 500,000 Finns. The study participants include patients with acute and chronic diseases, healthy volunteers, and population collections. R12 consists of 520,210 individuals (55% females and 45% males).

### FinnGen Ethics Statement

Study subjects in FinnGen provided informed consent for biobank research, based on the Finnish Biobank Act. Alternatively, separate research cohorts, collected prior the Finnish Biobank Act came into effect (in September 2013) and the start of FinnGen (August 2017), were collected based on study-specific consents and later transferred to the Finnish biobanks after approval by Fimea (Finnish Medicines Agency), the National Supervisory Authority for Welfare and Health. Recruitment protocols followed the biobank protocols approved by Fimea. The Coordinating Ethics Committee of the Hospital District of Helsinki and Uusimaa (HUS) statement number for the FinnGen study is Nr HUS/990/2017.

The FinnGen study is approved by Finnish Institute for Health and Welfare (permit numbers: THL/2031/6.02.00/2017, THL/1101/5.05.00/2017, THL/341/6.02.00/2018, THL/2222/6.02.00/2018, THL/283/6.02.00/2019, THL/1721/5.05.00/2019 and THL/1524/5.05.00/2020), Digital and population data service agency (permit numbers: VRK43431/2017-3, VRK/6909/2018-3, VRK/4415/2019-3), the Social Insurance Institution (permit numbers: KELA 58/522/2017, KELA 131/522/2018, KELA 70/522/2019, KELA 98/522/2019, KELA 134/522/2019, KELA 138/522/2019, KELA 2/522/2020, KELA 16/522/2020), Findata permit numbers THL/2364/14.02/2020, THL/4055/14.06.00/2020, THL/3433/14.06.00/2020, THL/4432/14.06/2020, THL/5189/14.06/2020, THL/5894/14.06.00/2020, THL/6619/14.06.00/2020, THL/209/14.06.00/2021, THL/688/14.06.00/2021, THL/1284/14.06.00/2021, THL/1965/14.06.00/2021, THL/5546/14.02.00/2020, THL/2658/14.06.00/2021, THL/4235/14.06.00/2021, Statistics Finland (permit numbers: TK-53-1041-17 and TK/143/07.03.00/2020 (earlier TK-53-90-20) TK/1735/07.03.00/2021, TK/3112/07.03.00/2021) and Finnish Registry for Kidney Diseases permission/extract from the meeting minutes on 4th July 2019.

The Biobank Access Decisions for FinnGen samples and data utilized in FinnGen Data Freeze 11 include: THL Biobank BB2017_55, BB2017_111, BB2018_19, BB_2018_34, BB_2018_67, BB2018_71, BB2019_7, BB2019_8, BB2019_26, BB2020_1, BB2021_65, Finnish Red Cross Blood Service Biobank 7.12.2017, Helsinki Biobank HUS/359/2017, HUS/248/2020, HUS/430/2021 § 28, § 29, HUS/150/2022 § 12, § 13, § 14, § 15, § 16, § 17, § 18, § 23, § 58, § 59, HUS/128/2023 § 18, Auria Biobank AB17–5154 and amendment #1 (August 17 2020) and amendments BB_2021 – 0140, BB_2021 – 0156 (August 26 2021, Feb 2 2022), BB_2021 – 0169, BB_2021 – 0179, BB_2021 – 0161, AB20–5926 and amendment #1 (April 23 2020) and itś modifications (Sep 22 2021), BB_2022 – 0262, BB_2022 – 0256, Biobank Borealis of Northern Finland_2017_1013, 2021_5010, 2021_5010 Amendment, 2021_5018, 2021_5018 Amendment, 2021_5015, 2021_5015 Amendment, 2021_5015 Amendment_2, 2021_5023, 2021_5023 Amendment, 2021_5023 Amendment_2, 2021_5017, 2021_5017 Amendment, 2022_6001, 2022_6001 Amendment, 2022_6006 Amendment, 2022_6006 Amendment, 2022_6006 Amendment_2, BB22–0067, 2022_0262, 2022_0262 Amendment, Biobank of Eastern Finland 1186/2018 and amendment 22§/2020, 53§/2021, 13§/2022, 14§/2022, 15§/2022, 27§/2022, 28§/2022, 29§/2022, 33§/2022, 35§/2022, 36§/2022, 37§/2022, 39§/2022, 7§/2023, 32§/2023, 33§/2023, 34§/2023, 35§/2023, 36§/2023, 37§/2023, 38§/2023, 39§/2023, 40§/2023, 41§/2023, Finnish Clinical Biobank Tampere MH0004 and amendments (21.02.2020 & 06.10.2020), BB2021–0140 8§/2021, 9§/2021, § 9/2022, § 10/2022, § 12/2022, 13§/2022, § 20/2022, § 21/2022, § 22/2022, § 23/2022, 28§/2022, 29§/2022, 30§/2022, 31§/2022, 32§/2022, 38§/2022, 40§/2022, 42§/2022, 1§/2023, Central Finland Biobank 1–2017, BB_2021 – 0161, BB_2021 – 0169, BB_2021 – 0179, BB_2021 – 0170, BB_2022 – 0256, BB_2022 – 0262, BB22–0067, Decision allowing to continue data processing until 31st Aug 2024 for projects: BB_2021 – 0179, BB22–0067,BB_2022 – 0262, BB_2021 – 0170, BB_2021 – 0164, BB_2021 – 0161, and BB_2021 – 0169, and Terveystalo Biobank STB 2018001 and amendment 25th Aug 2020, Finnish Hematological Registry and Clinical Biobank decision 18th June 2021, Arctic biobank P0844: ARC_2021_1001.

### Phenotypic definitions in FinnGen and UK Biobank

Exact definitions of ICD[8,9,10] diagnoses, medications, and procedures for all FinnGen phenotypes are publicly available at https://risteys.finregistry.fi/ using the tag names listed in the summary below. Detailed definitions of UKBB phenotypes are included in the Supplementary info (“Detailed Phenotype Descriptions”).

To create a large but specific set of autoimmune hypothyroidism individuals, we first collected all individuals with a diagnosis of hypothyroidism (most commonly ICD10 E03.9) and who also had 3 + refills of levothyroxine. From this set of individuals, we then excluded those who had thyrotoxicosis, thyroidectomy, iodine deficiency, and individuals with pituitary diseases likely to have central hypothyroidism of non-autoimmune origin. Detailed descriptions of the FinnGen phenotype can be found at https://risteys.finregistry.fi/endpoints/E4_HYTHY_AI_STRICT. The UKBB phenotype was created using similar criteria and detailed coding can be found in the Supplementary information.

To discriminate autoimmune versus thyroid loci we created a set of individuals with autoimmune disease but who did not have hypothyroidism or autoimmune hyperthyroidism. The complete list of selected autoimmune diseases is listed at https://risteys.finregistry.fi/endpoints/AUTOIMMUNE. For the phenotype AUTOIMMUNE_NONTHYROID in both FinnGen and UKBB, from the selected individuals with autoimmune disease, we then excluded those with thyroidectomy, use of levothyroxine or carbimazole, any individuals in the strict autoimmune hypothyroidism cases described above, and those with autoimmune hyperthyroidism (ICD-10: E05[0|9] ICD-9: 2420).

### Meta-analysis and definition of LD-independent associations

Genotyping, QC and imputation were performed as described in detail in the FinnGen resource paper (Kurki et al. 2023) In FinnGen r8 (available at https://www.finngen.fi/en/access_results), Regenie version 2.2.4 was used with sex, age, 10 principal components (PCs) of ancestry and genotyping batch included as covariates. UK Biobank analysis was performed using the pipeline implemented for the Pan UKBB project (https://pan.ukbb.broadinstitute.org/) using SAIGE with age, sex, age * sex, age^2^, age^2^ * sex, and 10 ancestry PCs. Meta-analysis of the FinnGen r12 and UKBB European ancestry subset (n = 420531 after QC and population clustering described on the Pan UKBB website) was performed using inverse-variance weighted meta-analysis.

As high-resolution fine-mapping algorithms have not been shown to be fully reliable in the context of meta-analyses particularly when performed using different genotyping and imputation techniques, we opted conservatively to flag only index variants representing the most significantly associated variant in confidently LD independent loci. LD independent associations were determined as follows: starting with the most significant association with p < 5e-8 on each chromosome, around each genome-wide significant variant, a +/− 2Mb window was screened. A dynamic LD threshold for each association is defined as T = min (0.1, r_5_) where r_5_ is defined as the r^2^ value at which the expected residual chi-square would be 5.0. Secondary associations were counted only if they were genome-wide significant and r^2^ to any more significant listed association was < T. Because of potential inaccuracy of low values of r^2^, at particularly strong associations where T < 0.02 (that is, residual association signal may exist even at very low values of r^2^) secondary associations were not defined within 1Mb of such signals. Nearby signals were confirmed as independent using full conditional analysis in FinnGen on top signals using Regenie and owing to the limited accuracy of pairwise LD inference beyond two signals, we only report here two signals within 1 Mb with the exception of a handful of examples where three signals within 1 Mb were all conditionally independently associated at genome-wide significance in FinnGen.

### Bayesian classification of association results

Linemodels (https://github.com/mjpirinen/linemodels) was used to explore the existence of and classify individual variants into, clusters based on bivariate effects. Using as input the (beta,se) pairing from two GWAS analyses for a set of variants, linemodels probabilistically clusters variants into groups, providing both a likelihood of each number of groups and posterior probability of assignment to each group. Linemodels consist of three parameters: scale (the magnitude of effect), slope (the multiplicative relationship between the effects on each phenotype) and correlation (the expected consistency with the expected values). We set the scale parameter to 0.6, such that 95% of the effect sizes are within two times the scale parameter for all groups. We also chose a correlation parameter of 0.99 to permit modest deviation from the exact best-fit slope. Models with 1 or 2 slopes were fit using the EM-algorithm implemented in linemodels and likelihood-ratio test used to compare models. For the comparisons involving AInonT and SKIN, one of the slopes was set to 0 in order to capture only those variants that belong to AIHT, while the other slope was optimized with an EM-algorithm. The slopes for shared groups were 0.447 for AInonT, and − 0.384 for SKIN. When running linemodels for TSH, both slopes were allowed to optimized (since AIHT is so common, purely autoimmune associations will induce a residual effect on TSH population-wide) and were found to be 0.137 and 0.964. After optimizing slopes, we used an iterative Gibbs Sampler to assign group probabilities.

### PGS-Phenome-Wide Association Study

We conducted a Phenome-Wide Association Study (PheWAS), investigating the associations between a Polygenic Score (PGS) for Hypothyroidism, derived from the UKBB data (Weissbrod et al. 2022), and 4,739 phenotypes from the FinnGen biobank (R11). We excluded 149 endpoints with fewer than 50 cases from the analysis. We applied logistic regression with LDLT-decomposition using the ‘*fastglm*’ R package (https://CRAN.R-project.org/package=fastglm). The association between the standardized PGS vector and each endpoint was adjusted for Sex, Age, Age^2^, the first six principal components, and genotyping array features:

Our primary focus was , with other factors included to address potential residual confounding, providing no additional significant information for our study. For gender-specific traits was excluded. We set a Bonferroni threshold at a *p*-value < 1.05*10^− 5^ (0.05/4,739) to handle multiple comparisons problem.

In addition to the described experiment, we conducted two modified PheWASs to further explore our findings. The first, an “exclusion-PGS-PheWAS” (Fritsche et al. 2018), aimed to determine if the secondary trait associations with the Hypothyroidism score were influenced by overlapping samples between the hypothyroidism endpoint (E4_HYTHY_AI_STRICT) and studied phenotype. For this, we removed hypothyroidism cases from the analysis and conducted PheWAS on such filtered cohorts. The second study, a “noMHC-PGS-PheWAS”, examined whether the associations we discovered were driven by the presence of the MHC locus. In this study, we excluded variants located in the MHC region (6:28,510,120 – 33,480,577; GRCh38) from the PGS model and then assessed the phenome-wide associations using the modified polygenic score.

## Data Availability

Data will be available in a Google Cloud platform storage bucket upon publication.
